# A differential response to newt regeneration extract by C2C12 and primary mammalian muscle cells

**DOI:** 10.1186/s13395-015-0044-8

**Published:** 2015-06-11

**Authors:** Sarah Kawesa, Jason Vanstone, Catherine Tsilfidis

**Affiliations:** Ottawa Hospital Research Institute, Vision Research/Regenerative Medicine Program, 501 Smyth Road, Box 307, Ottawa, Ontario K1H 8L6 Canada; Department of Cellular and Molecular Medicine, University of Ottawa, 451 Smyth Road, Ottawa, Ontario K1H 8M5 Canada; Current address: Children’s Hospital of Eastern Ontario Research Institute, 401 Smyth Road, Ottawa, Ontario K1H 8L1 Canada

**Keywords:** Newt forelimb regeneration, Dedifferentiation, C2C12, Myoblasts, Myotubes

## Abstract

**Background:**

Dedifferentiation, a process whereby differentiated cells lose their specialized characteristics and revert to a less differentiated state, plays a key role in the regeneration process in urodele amphibians such as the red spotted newt, *Notophthalmus viridescens*. Dedifferentiation of fully mature tissues is generally absent in mammalian cells. Previous studies have shown that mouse C2C12 multinucleated myotubes treated with extract derived from regenerating newt forelimbs can re-enter the cell cycle, fragment into mononucleated cells, and proliferate. However, this response has been difficult to replicate.

**Methods:**

We isolated extract from early newt forelimb regenerates and assessed its effects on differentiation of proliferating primary and C2C12 myoblasts. We also treated fully differentiated primary and C2C12 myotube cultures with extract and assessed cell cycle re-entry and myotube fragmentation.

**Results:**

We have confirmed the results obtained in C2C12 cells and expanded these studies to also examine the effects of newt regeneration extracts on primary muscle cells. Newt extract can block differentiation of both C2C12 and primary myoblasts. Once differentiation is induced, treatment with newt extract causes cell cycle re-entry and fragmentation of C2C12 myotubes. Downregulation of p21 and muscle-specific markers is also induced. Primary myotubes also fragment in response to extract treatment, and the fragmented cells remain viable for long periods of time in culture. However, unlike C2C12 cells, primary muscle cells do not re-enter the cell cycle in response to treatment with newt extracts.

**Conclusions:**

Dedifferentiation of fully mature muscle occurs during regeneration in the newt forelimb to contribute cells to the regeneration process. Our study shows that extracts derived from regenerating newt forelimbs can induce dedifferentiation, cell cycle re-entry, and fragmentation of mouse C2C12 cells but can only induce fragmentation in primary muscle cells.

**Electronic supplementary material:**

The online version of this article (doi:10.1186/s13395-015-0044-8) contains supplementary material, which is available to authorized users.

## Background

Tissue regeneration is a complex process of replacing cells lost due to disease or injury [1, 2]. All eukaryotes have basic regenerative abilities such as wound closure and tissue repair. The most complex form of regeneration is known as epimorphic regeneration. This is mastered by the urodele amphibians (newts and axolotls). Epimorphic limb regeneration involves four major phases: wound healing, dedifferentiation, blastema formation, and redifferentiation [3]. It is believed that the dedifferentiation process is the key to regeneration [4, 5]. Through this process, differentiated cells re-enter the cell cycle and revert back to a progenitor phenotype [6–8] that can then proliferate and re-differentiate into the various tissues of the regenerated structure.

In mammals, dedifferentiation of fully differentiated muscle does not occur during the regeneration process. Satellite cells are the source of cells for regeneration of damaged mammalian muscle (see review [9]). Interestingly, recent studies show that satellite cells are also the source of cells for muscle regeneration in the axolotl, a urodele amphibian known for its tremendous regenerative power [10]. Newts, however, dedifferentiate mature muscle cells during the regeneration process [10]. Downregulation of p53 [11] and activation of ERK [12] are critical to the dedifferentiation process.

Numerous *in vitro* studies have been conducted with the newt A1 muscle cell line to study the dedifferentiation process. Studies with newt A1 myotubes show that serum stimulation results in cell cycle re-entry [13–15]. This early dedifferentiation event is not seen in mammalian myotubes. However, some dedifferentiation studies have been successful in the mammalian C2C12 muscle cell line [16]. C2C12 myotubes undergo dedifferentiation in culture in response to fusion with newt cells [17], or treatment with extracts from newt forelimb regenerates [18]. While the C2C12 cell line is ideal for studying the reversal of differentiation, recent studies have found that the cell line has a deletion in the INK4a/ARF locus [19]. The INK4a/ARF gene encodes p16, a cyclin-dependent kinase inhibitor, and p19, alternate reading frame (ARF) that controls p53 function [20]. Since established cell lines can become altered with time, in order to further characterize dedifferentiation in mammalian cells, primary muscle cell lines should be used. Studies that have attempted to induce dedifferentiation in primary myotubes have found that in normal cells, cell cycle re-entry does not occur [21, 22]. In contrast to the studies by Schneider et al. [23] that showed cell cycle re-entry in myotubes derived from an immortalized Rb-deleted cell line, two studies have shown that Rb removal alone cannot cause differentiated myotubes to re-enter the cell cycle in a primary cell line [22, 21]. Pajcini et al. [19], however, found that inactivation of Rb and p19^ARF^ in primary myotubes resulted in cell cycle re-entry. In addition, inactivation of Rb and p19^ARF^ led to downregulation of differentiation markers including muscle creatine kinase (MCK), myosin heavy chain (MHC), and MRF4 and upregulation of cyclin D1 and cyclin E. This study indicated that p19^ARF^ might be the factor that impedes cell cycle re-entry in terminally differentiated muscle cells.

A previous study has suggested that newt extract derived from the early limb regenerate has the ability to induce cell cycle re-entry and dedifferentiation in mammalian myotubes [18]. This would suggest that mammalian cells may be capable of undergoing dedifferentiation, and that a factor(s) present in the newt extract provides the trigger to initiate the response. However, the studies were conducted in C2C12 cells, and so the question remains whether this is a global capability common to all mammalian muscle cells or a selective response by the INF4a/ARF-deleted C2C12 cells. The current study compares the responses of C2C12 and primary myotubes to newt regeneration-derived extracts and determines whether there is something specific to newt extract that might inactivate mammalian cell cycle checkpoints and allow dedifferentiation.

## Methods

### Animals

Adult *Notophthalmus viridescens* red spotted newts were purchased from Charles D. Sullivan Co. Inc. (Nashville, TN). Animals were housed at 22 °C in large aerated tubs with running dechlorinated water and fed weekly on live blackworms. All experimental procedures were performed under anesthesia by immersion in buffered 0.1 % tricaine methanesulphonate (MS222, Sigma). Experimental protocols were approved by the University of Ottawa Animal Care and Veterinary Service.

### Preparation of newt extract

Under anesthesia, an initial bilateral amputation was done above the wrist. Forelimbs were re-amputated at 5 days after the initial amputation 1 mm proximal to the initial amputation site and the regenerated tissues were collected. The forelimbs were then re-amputated 3 days later, and again after 1 day, as shown in Additional file 1. The sampled tissues were immediately snap-frozen in liquid nitrogen and stored at −80 °C. The primary newt extract (1 °) was prepared from first-time amputated tissues. Secondary extract (2 °) was prepared from animals that were previously amputated, allowed to regenerate for 1 or 3 months, and then re-amputated. As with the primary extract, tissues were again collected at 5, 3, and 1 days after the amputation. Extract was made from the pooled tissue samples of approximately 30 newts with slight variations to the protocol of McGann et al. [18]. Control extract was made from the intact forelimb tissues. The frozen tissues were thawed and placed in 10 ml High Glucose Dulbecco’s Modified Eagle Medium (DMEM; Hyclone) supplemented with a Protease Inhibitor Cocktail (Roche). One tablet of PIC was dissolved in 1.5 ml of distilled water. One milliliter of the dissolved solution was added to 9 ml of DMEM. The tissues were homogenized for 5–10 min using a VDI 12 hand homogenizer (VWR), and sonicated for 1–3 min with a Misonix XL2000 sonicator. The homogenate was spun for 25 min at 2000 × *g* at 4 °C. The supernatant was then placed in a fresh tube and spun for 60 min at 100,000 × *g* at 4 °C using an ultracentrifuge. The supernatant was filter sterilized through a 0.4-μm filter. The extract concentration was determined with a BCA protein assay kit (Bio-Rad) and stored in 0.5 ml aliquots at −80 °C.

#### Fractionation of extract

One hundred microliters of crude extract at 2.5 mg/ml was added to a Vivaspin 500 30 kDa molecular weight cut off (MWCO) polyethersulphone (PES) column (Sartorius). The samples were spun for 30 min at 16,200 × *g*. The >30 kDa fraction that was retained on the filter was resuspended in 25 μl. The concentration of the separated fractions (≤30 kDa and ≥30 kDa) was examined with a BCA protein assay kit (Bio-Rad) and stored at −80 °C. All procedures for preparation of extract were carried out at 4 °C.

### Differentiation of C2C12 cells

C2C12 cells were obtained from ATCC. Myoblasts were grown to confluency on 100-mm plates (Corning Inc.) in GM. To induce differentiation into multinucleated myotubes, the cells were switched from GM with 10 % FBS to differentiation medium (DM) containing 2 % horse serum (Sigma).

### Isolation and differentiation of primary myoblasts

Primary myoblasts were isolated from the hindlimb muscle of 4–6-week-old mice according to the protocol of Springer et al. [24]. Myoblasts were grown in Ham’s F-10 medium (Sigma), supplemented with 20 % FBS, 4X pen/strep, 1X fungizone (Gibco), 50 μg/ml gentamicin (Gibco) and 10 ng/ml basic fibroblast growth factor (Sigma). Cells were grown on collagen-coated plates. Once the primary myoblasts reached approximately 70 % confluency, they were switched to DM to induce myotube formation.

### Effect of newt extract on myoblast proliferation

#### Flow cytometry analysis

Semi-confluent myoblasts that were grown in GM for 48 h with or without extract were trypsinized and washed once in 10 % FBS-DMEM. Myoblasts were then washed twice in PBS supplemented with 1 mM EDTA (PBSE) and then fixed in 1 ml of PBSE by the drop-wise addition of 2 ml of 80 % ethanol, pre-chilled to −20 °C. The samples were stored at −20 °C for a minimum of 2 h, washed once in PBSE, resuspended in DNA content staining buffer (1.1 % citrate buffer, 10 μg/μl propidium iodide (Sigma), 0.1 mg/ml RNase) and incubated for 30 min at 37 °C before being analyzed on a Beckman-Coulter flow cytometer using the Expo 32 software package.

#### Hemocytometer analysis

Myoblasts were plated on 100-mm plates (Corning Inc.) in GM until they became semi-confluent. Cells were then trypsinized and resuspended in GM. The same number of cells (approx. 1.5 × 10^4^) was plated either in the presence or absence of newt extract. After 48 h in culture, the cells were re-counted using a hemocytometer.

### Effects of newt extract on muscle differentiation

Myoblasts were kept in GM until they were 70 % confluent for primary cells and 100 % for C2C12 cells. The myoblast cultures were then treated with 0.3 mg/ml of newt blastema extract in DM or GM for 4 days followed by immunostaining with MF20 antibody (1:50), which stains myosin heavy chain (MHC). The control cells were untreated, treated with heat-inactivated extract or extract derived from intact forelimb tissue.

### Purification of myotubes

#### AraC treatment

In order to remove contaminating myoblasts from the myotube cultures, differentiated myotubes were transferred from DM to GM with 10 μM arabinosylcytosine (AraC; Sigma) for 48 h. After treatment with AraC, the cells were washed in phosphate buffered saline (1X PBS) and transferred back into DM for a 24-h recovery period prior to further treatments.

#### Cell filtration

In this alternative method for removing contaminating myoblasts, day 4 differentiated myotubes were passed through a 100-μM nylon mesh cell strainer (Fisher). Large clumps of cellular debris were retained on the filter. The eluate was passed through a 40-μM mesh to get rid of most of the contaminating myoblasts. Myotubes that were retained on the filter were resuspended in medium and re-plated onto culture dishes.

### Tracking of differentiated cells

Proliferating C2C12 myoblasts cultured in 10 % FBS were co-transfected with both 5-μg pCMV-EGFP/RFP (Addgene) and 5-μg MCK-Cre (Addgene) plasmids using Exgen500 reagent (Fermentas). Cells were maintained under G418 selection. When the cells reached confluency, the medium was switched to 2 % HS to induce differentiation.

In a separate experiment, tracking of differentiated myotubes was accomplished by microinjection of a GFP plasmid (pEGFP-Cl, Addgene, 100 ng/μl) directly into the myotubes. The plasmid was loaded into a FemtoTip needle (Eppendorf) using a microloader. For the injection, an injection pressure of 150 hPa, a maintenance pressure of 50 hPa, and an injection time of 0.1 s was used.

### Treatment of myotube cultures with newt extract

Myotubes were treated with 0.1 mg/ml or 0.3 mg/ml of primary (1 °) or secondary (2 °) newt extract in 2 % horse serum or 10 % fetal bovine serum for 3–10 days. In the presence of newt extract, 1 mM bromodeoxyuridine (BrdU; Invitrogen) was added to the cells. Control cells were untreated or treated with heat-inactivated newt extract.

### Detection of apoptotic cells

Apoptotic cells were identified using terminal deoxynucleotidyl transferase (TdT)-mediated dUTP nick end labeling (TUNEL) using the ApopTaq Fluorescein or ApopTaq Red *In situ* Apoptosis Detection Kit (Chemicon) according to the manufacturer’s instructions. C2C12 control and extract-treated cells were pre-fixed in 1 % PFA in PBS (pH 7.4) for 10 min at RT. The cells were post-fixed at −20 °C for 5 min in 2:1 ethanol:acetic acid. After the washes, the fixed cells were incubated with the kit reagents, followed by MF20 antibody staining, after which they were examined by fluorescence microscopy.

### Microinjection of myotubes with newt extract

#### Preparation and injection of cells

Day 3 myotubes were purified using filtration (as described above). Filtered C2C12 cells were plated on 35-mm non-coated plates, or plates coated with 0.8 % sodium hyaluronate or 0.4 % collagen. Primary myotubes were plated on either 0.8 % sodium hyaluronate or 0.4 % collagen. Microinjection was performed using a micromanipulator and a Zeiss inverted microscope. Myotubes were microinjected with 1:1 ratio of extract (at approx. 4 mg/ml) and 100 ng/μl of GFP plasmid (pEGFP-Cl, Addgene). The extract/plasmid mixture was loaded into a FemtoTip needle and the injection parameters were as described above. From 25 to 60 myotubes were microinjected with the extract/plasmid cocktail. Right after the injection, the cells were transferred to 10 % FBS for 3 or 4 days. Control myotubes were microinjected with DMEM or MEF extract and GFP plasmid.

### Immunocytochemistry

Control or extract-treated cultures were washed with 1X PBS before fixation. Cells were fixed in 4 % paraformaldehyde (PFA) for 5 min at room temperature, followed by 3 washes of 1X PBS for 5 min each. The primary antibodies incubated with the cells included the following: monoclonal antibody BrdU, 1:500 dilution (Molecular Probes); monoclonal antibody Ki-67, 1:200 dilution (Sigma); monoclonal antibody p21, 1:50 (BD Biosciences, Pharmingen); polyclonal antibody MF20, 1:50 (Santa Cruz Biotechnology) and monoclonal antibody GFP, 1:200 (Invitrogen). The secondary antibodies were goat anti-mouse Alexa Fluor 488 (Invitrogen) and anti-rabbit Cy3 (Jackson Immuno Research, 1:400 dilutions for both). Fluorescence was observed on a Zeiss Axiovert or Nikon inverted microscope.

For the BrdU assay, several additional steps were performed prior to the addition of the primary antibody. Cells were blocked in 5 % goat serum (Jackson Immuno Research), 0.3 % Triton X-100 (Sigma) in PBS for 30 min at room temperature. The cells were then incubated in 1 N HCl (Sigma) for 10 min on ice, followed by 2 N HCI for 10 min at room temperature and for 20 min at 37 °C, and washed in PBS (3X, 5 min each). To buffer the cells, 0.1 M borate buffer (ACP Chemical Inc) was added for 12 min at room temperature.

### Fragmentation and proliferation assay

Fragmentation was assessed by following newt-extract-treated myotube cultures with a Zeiss live imaging microscope. C2C12 and primary cultures were differentiated for 2 days followed by AraC treatment as described above for 2 days. The cells were then placed back into DM for 1 day followed by newt extract for 3 days at a concentration of 0.3 mg/ml in DM. Cells were followed with the Zeiss live imaging microscope and photographed every 15 min. Fragmentation and proliferation were also assessed in both primary and C2C12 GFP myotubes that were microinjected with a 1:1 ratio of GFP plasmid (100 ng/μl) and newt extract.

### Real-time polymerase chain reaction (RT-PCR) analysis for myogenin

Total RNA was isolated from extract-injected and control myotubes at 96 h after injection of extract/GFP or DMEM/GFP. RNA was isolated using the RNeasy minikit (Qiagen). RNA concentration was determined on a spectrophotometer and the quality of RNA was verified by electrophoresis on a 1 % agarose gel. Equal amounts of RNA were reverse transcribed with oligo dT-primers to give complementary DNA (cDNA) using a cDNA synthesis kit (Bio-Rad). RT-PCR was conducted using the SYBR green system (Bio-Rad). Gene-specific primers for myogenin were designed using Primer Software Version 3. PCR was performed using primers 5′-GCAATGCACTGGAGTTCG-3′ and 5′-ACGATGGACGTAAGGGAGTG-3′ on the Bio-RAD iCycler at 95 °C for 5 min followed by 35 cycles of 95 °C for 30 s, 58 °C for 30 s, 72 °C for 30 s. Expression profiles were normalized against GAPDH (primers 5′-TCGGTGTGAACGGATTTG-3′ and 5′-GGTCTCGCTCCTGGAAGA-3′). Expression levels were calculated using the 2-ΔΔCT method. The quality of the PCR products was examined by electrophoresis on a 1 % agarose gel.

### Statistics

The data are presented as means ± standard deviation. The statistical calculations were done using GraphPad Prism 5. Differences between control and treated samples were analyzed using a two-way ANOVA or a Student *t*-test. *P* values less than 0.05 were considered significant.

## Results

### The effect of newt extract on myoblast proliferation

Newt extract was isolated (see Additional file 1) using slight modifications of the published protocol [18]. Since a single batch of extract was not sufficient to conduct all the experiments described here, we tested various isolates of extract. We also compared the efficacy of primary extract (isolated from newly amputated tissues) and secondary extract (resulting from the re-amputation of 1–3-month regenerated tissues). We reasoned that perhaps regenerating tissues (used to generate the secondary extract) might be “primed” by the regeneration process and might produce a more effective and more “potent” extract.

Before testing the effects of the extract on fully differentiated muscle cells, we assessed the effects on proliferation of myoblasts. Cell proliferation was analyzed using flow cytometry in C2C12 semi-confluent cells treated with newt extract. Equal numbers of cells were plated in both control and extract-treated cultures. After 48 h, the cells were harvested for flow cytometry analysis (Additional file 2A, B). There were no apparent effects on cell cycle in myoblasts that were treated with 1° newt extract at 0.3 mg/ml in comparison to the control. Cell proliferation was also analyzed using a hemocytometer. Semi-confluent myoblasts were plated with the same number of cells in 1 ml of growth medium (GM) either in the presence or absence of newt extract. After 48 h in culture, the cells were re-counted using a hemocytometer. Again, there was no difference seen between the control and the extract-treated cultures (Additional file 2C). This indicates that the newt extract does not increase proliferation in myoblast cultures.

### The effect of newt extract on myoblast differentiation

**Fig. 1:**
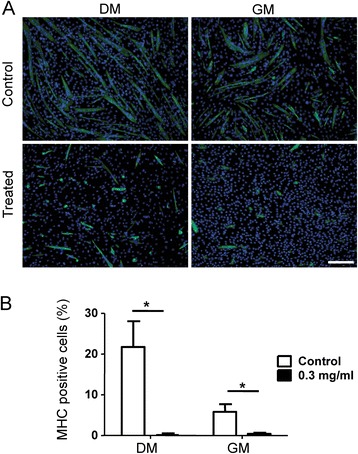
Inhibition of muscle differentiation in C2C12 cells treated with newt extract. **a** C2C12 myoblasts treated with 0.3 mg/ml of newt extract in either differentiation medium (DM) or growth medium (GM) showed impaired differentiation and reduced numbers of MHC-positive cells. **b** The percentage of MHC-positive cells was calculated by adding all MHC-positive cells (myotubes and myocytes) and dividing by the total number of cells. The percentage of MHC-positive cells was reduced in extract-treated cells in both DM and GM. *Scale bar* = 200 μm. *p < 0.0001

Myogenic differentiation is typically studied *in vitro* by the fusion of myoblasts into multinucleated myotubes. To examine the effect of newt extract on myoblast differentiation, the newt extract at a concentration 0.3 mg/ml was added to subconfluent C2C12 myoblasts, and when these cultures reached confluence, they were transferred to differentiation medium (DM) or GM for 4 days; then the cells were analyzed for their ability to undergo myogenic differentiation. When confluent myoblasts were cultured without newt extract in both DM and GM for 4 days, the cells underwent differentiation to form multinucleated myotubes (Fig. 1a). Cells grown in differentiation medium formed larger and more numerous myotubes, as might be expected, but even in growth medium, confluent myoblasts exited the cell cycle and formed myosin heavy chain (MHC)-positive myotubes. In contrast, the newt extract-treated cells formed fewer multinucleated myotubes than the control (Fig. 1a) and they were much smaller in size. A significant decrease in the expression of myosin heavy chain (MHC) was observed in the extract-treated culture compared to the control in GM and DM (Fig. 1b). This suggests that newt extract has effects on myoblast differentiation and on myoblast fusion into multinucleated C2C12 myotubes.

**Fig. 2:**
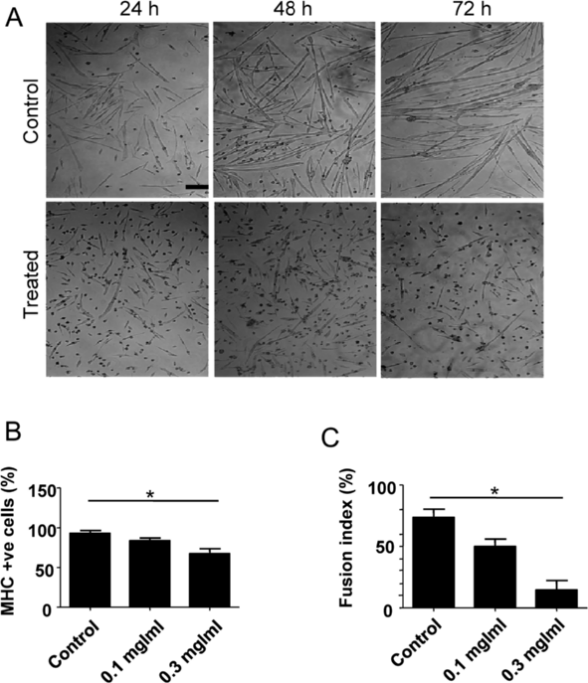
Inhibition of differentiation in primary myoblasts treated with newt extract. **a** Primary myoblasts treated with 0.3 mg/ml of newt extract for 72 h showed reduced numbers of myotubes. **b** The percentage of myosin heavy chain (MHC)-positive cells (see Fig. 3b for method of calculation) was reduced in extract-treated cells. **c** Fusion index was reduced in primary myoblasts treated with 0.1 mg/ml or 0.3 mg/ml of extract. The degree of reduction was related to the concentration of the extract. Fusion index was calculated as the number of nuclei within multinucleated myosin-positive cells divided by the total number of nuclei × 100. *Scale bar* = 200 μm. *p < 0.05

The effect of newt extract on primary myoblast differentiation was also assessed. Primary myoblasts were grown to 70 % confluency in GM, and were then switched to DM in the presence or absence of newt extract. Within 24 h, myotube formation was observed in the control cultures (Fig. 2a). Myotubes increased in number and in size with time. In the extract-treated cultures, however, there was a significant decrease in the number of myotubes (Fig. 2a). This was manifested as a reduction in MHC-positive cells in comparison to the control cells (Fig. 2b), but this effect was not as pronounced as with the C2C12 cells. Myoblast fusion, on the other hand, was severely affected (Fig. 2c). This suggests that, unlike C2C12 cells in which both myoblast differentiation and fusion are severely affected by treatment with newt extract, primary cells show more pronounced effects on myoblast fusion, and less on differentiation. The effects appear to be concentration-dependent, since 0.1 mg/ml of extract had a smaller effect than 0.3 mg/ml (Figs. 2b, c). Importantly, the presence of newt extract in the culture did not appear to affect the proliferation of subconfluent myoblasts (either positively or negatively), nor did it impact cell survival (or apoptosis).

### Cell cycle re-entry in AraC- and extract-treated C2C12 myotubes

McGann et al. [18] showed that mammalian C2C12 myotubes treated with newt extract could re-enter the cell cycle and fragment into mononucleated cells. Our first aim was to replicate the results in C2C12 myotubes and then to extend them into the study of primary cells. C2C12 myoblasts were grown to confluency and induced to differentiate into multinucleated myotubes. In order to remove contaminating myoblasts from the cultures and obtain purer myotube populations, cultures were treated with AraC, a cytosine analog that incorporates into replicating DNA, prevents the cell from dividing, and leads to cell death. Subsequently, the differentiated cells were treated with newt extract for 3 days in the presence of BrdU in either GM or DM. We included GM in the extract treatments because we reasoned that if the cells were induced to re-enter the cell cycle, they would need supporting nutrients to prevent them from undergoing apoptosis. Control cells were treated with heat-inactivated extract or extract derived from intact newt forelimb tissues. Cells were then fixed and stained with anti-BrdU antibodies to assess cell cycle re-entry, and MF20 to label MHC-positive myotubes.

**Fig. 3:**
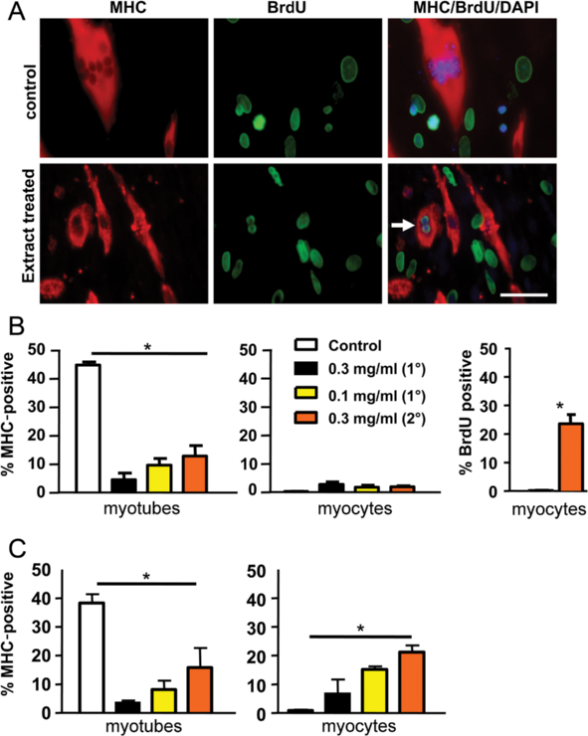
Cell cycle re-entry in C2C12 myotube cultures. Myotube cultures were treated with AraC to eliminate cycling myoblasts, and then treated with various concentrations of 1° or 2° newt extract in the culture medium. **a** *Arrow* in treated culture identifies MHC-positive myotube that was also positive for BrdU. *Scale bar* = 100 μm. **b** Following extract treatment in GM, the percentage of MHC-positive cells was determined (number of myotubes divided by total number of cells for *left panel* and number of myocytes divided by total number of cells for *middle panel*). Extract treatment led to significant reductions in myotubes and small increases in myocytes. In cultures treated with 0.3 mg/ml of secondary (2 °) extract, 23 % of MHC-positive myocytes incorporated BrdU. **c** Similar to (**b**), extract treatment in DM not only led to a reduction in myotubes but also led to a significantly increased number of MHC-positive mononucleated myocytes. *p < 0.0001

Differentiated C2C12 cells are capable of re-entering the cell cycle following treatment with newt extract at a concentration of 0.3 mg/ml (Fig. 3a). Interestingly, cell cycle re-entry was almost exclusively seen in myocytes (mononucleated MHC-positive cells) or very small myotubes (two nuclei). Cell cycle re-entry in MHC-positive cells was never observed in control cultures. Notably, there was a significant decrease in multinucleated myotubes in cultures treated with newt extract in both GM (Fig. 3b) and DM (Fig. 3c), and this was accompanied by a significant increase in mononucleated myocytes when cells were treated in differentiation medium (Fig. 3c), suggesting that some of the decrease in myotube numbers may have resulted from fragmentation of multinucleated myotubes into mononucleated myocytes. The effect was dependent on the concentration of extract used, and the source of the extract. Not all batches of extract were equally effective. In fact, cell cycle re-entry was only induced in approximately 1/3 of the extracts we isolated. Coomassie blue staining of Western blots comparing four isolates revealed slight differences in the presence and concentrations of the proteins present in the different extracts (see Additional file 3). Interestingly, the most potent effects on fragmentation and cell cycle re-entry were obtained with 2 ° extract (obtained from newts that had undergone regeneration and were then re-amputated). In the experiment above, following treatment with 2 ° extract in GM, 23 % of MHC-positive myocytes were also positive for BrdU (Fig. 3b).

### No cell cycle re-entry in AraC- and extract-treated primary myotubes

Primary myoblasts underwent the same protocol of differentiation, AraC and extract treatment as C2C12 cells. Cell cycle re-entry was assessed by BrdU incorporation. In primary muscle cells, cell cycle re-entry was almost never observed in MHC-positive multinucleated or mononucleated cells treated with newt extract. In multiple replicates of the study, only a single myotube showed BrdU incorporation in one of its multiple nuclei (Additional file 4). In this case, it is probable that a dividing myoblast incorporated BrdU before fusing with an existing myotube. In addition, a significant decrease in MHC-positive myotubes was observed in cultures treated with newt extract. This was not accompanied by an increase in MHC-positive mononucleated cells, which might indicate that the decrease in myotubes resulted from cells dying and lifting off the plates.

### Combined AraC and extract treatment induces apoptosis in myotubes

Since the loss of myotubes seen in Fig. 3b, c could not solely be accounted for by fragmentation into myocytes, it is also possible that newt extract can induce apoptosis of myotubes. In fact, we noted a significant loss of large myotubes in extract-treated cultures. We conducted terminal dUTP nick end labeling (TUNEL) analysis in order to detect apoptotic cells. AraC-treated myotubes that were also treated with newt extract in GM showed a significant increase in TUNEL positive nuclei in comparison to the controls (Additional file 5A). This effect was dependent on the concentration of extract used and on the source of extract (with some batches showing increased toxicity, data not shown).

To further assess if the effect was being caused by newt extract or AraC (or both), we conducted a comparison between AraC- and non-AraC-treated myotubes that were also treated with newt extract. In the presence of newt extract, there appeared to be fewer and smaller myotubes, but only in the AraC-treated cultures (Additional file 5B). In other words, it appears that AraC treatment in combination with extract treatment induced the death of larger myotubes but appeared to spare many of the smaller myotubes with reduced numbers of nuclei. Possible reasons for this are discussed below.

### Genetically labeled myocytes incorporate BrdU following extract treatment

**Fig. 4:**
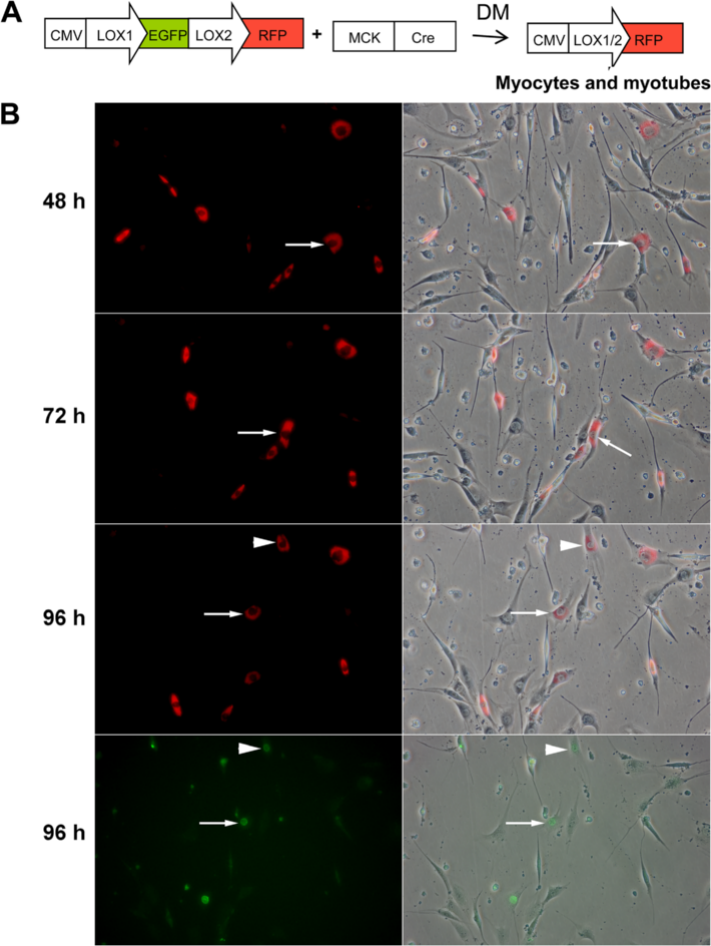
Further evidence of cell cycle re-entry in C2C12 myotube cultures. **a** Schematic diagram of plasmid vectors used to label cells. With these vectors, myoblasts express GFP. Upon differentiation, cells express MCK, leading to the expression of Cre and the excision of the GFP gene. Differentiated myotubes and myocytes are thus labeled with RFP. **b** BrdU was added to the cultures at 72 h and cells were fixed at 96 h and stained for BrdU incorporation (*green*). *Arrow* and *arrowhead* identify the same myocytes in all four panels at 96 h, showing nuclear BrdU expression in the RFP-expressing cells. At the same time, the *bottom panels* also show cytoplasmic diffuse GFP expression in neighboring myoblasts

The above experiments showed cell cycle re-entry in C2C12 multinucleated myotubes treated with newt extract. In order to eliminate the possible explanation that BrdU-incorporating myoblasts were fusing with existing myotubes to give these results, or were exiting the cell cycle after incorporating BrdU, we used a two-plasmid labeling system to identify differentiated cells. To label cells, proliferating C2C12 myoblasts were co-transfected with two plasmids, pCMV-EGFP/RFP, and MCK-Cre [25]. The first plasmid encodes a loxP-flanked green fluorescent protein (GFP) gene driven by the cytomegalovirus (CMV) promoter, and a red fluorescent protein (RFP). With this system, myoblasts express GFP. During differentiation, MCK-Cre is activated leading to excision of the loxP-flanked GFP gene, resulting in expression of RFP in differentiated myocytes or multinucleated myotubes (Fig. 4a).

Following differentiation, the RFP-expressing cultures were treated with newt extract in DM. BrdU was added to the cells at 72 h (after they had exited the cell cycle and already expressed RFP). At 96 h, the cells were fixed and stained for BrdU. Time-lapse images showed RFP-expressing myocytes which subsequently incorporated BrdU (Fig. 4b). This experiment was performed in triplicate, and multiple wells were studied for each replicate; BrdU incorporation in RFP-expressing cells was only seen in the extract-treated cultures.

### Differentiation-specific muscle markers are downregulated following extract treatment

Dedifferentiation should not only involve cell cycle re-entry but should also involve the downregulation of muscle-specific markers such as myosin heavy chain (MHC). In order to study downregulation of MHC, C2C12 myoblasts were differentiated and the resulting cultures were filtered to remove contaminating myoblasts. Filtering involved the passage of myotubes through 100- and 40-μm mesh, which allowed the undifferentiated myoblasts to pass through but retained the myotubes on the filters. This method was used rather than AraC treatment to avoid the substantial apoptosis of myotubes seen above. The filtered myotubes were re-plated, and following 24 h of recovery, multinucleated myotubes were microinjected with a GFP plasmid to allow tracking of myotubes. Cultures were treated with newt extract in DM at a concentration of 0.3 mg/ml of medium. Cells were treated for 6 days in DM and then switched to GM for a further 4 days. The cells were then fixed and stained with GFP and MHC antibodies.

**Fig. 5:**
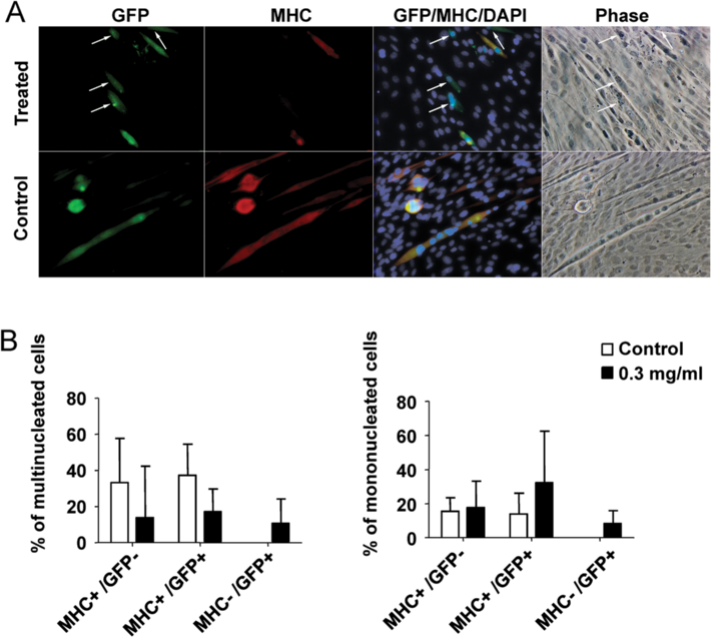
Evidence of downregulation of muscle-specific markers following treatment with newt extract. C2C12 myotubes were microinjected with a GFP tracer and subsequently treated with newt extract for 4 days in growth medium. They were then fixed and stained for myosin heavy chain (MHC). **a** The *arrow* identifies GFP-labeled cells that were not positive for MHC, suggesting that they had downregulated the MHC marker. Not all myotubes responded to the extract, as can be seen by GFP-labeled cells that were also positive for MHC. **b** Cell counts of multinucleated myotubes (*left panel*) and mononucleated myocytes (within the same cultures) showed that only extract-treated cultures had GFP-positive cells that were negative for MHC. This experiment was performed in triplicate, and multiple wells were counted for each replicate

We identified extract-treated C2C12 cells that were positive for GFP but negative for MHC (Fig. 5a). Since only multinucleated myotubes were injected with the GFP plasmid, the GFP^+^ cells that were negative for MHC could only have resulted from downregulation of the MHC marker. Since not all myotubes in the cultures were injected with the plasmid, counts were conducted to determine the proportion of cells that were expressing GFP or MHC. Notably, MHC−/GFP+ cells were only seen in extract-treated cultures (Fig. 5b). Control (non-extract-treated) GFP-injected cells were always positive for both MHC and GFP (Fig. 5a, b). Interestingly, not only did we identify myotubes that had downregulated the MHC marker, but we also found mononucleated myocytes that expressed GFP but not MHC. Since only multinucleated myotubes were injected with the GFP plasmid, this would suggest that fragmentation was also occurring in addition to downregulation of MHC.

### A decrease in P21 expression in extract-treated cultures

During muscle differentiation, the expression of p21 increases followed by cell cycle withdrawal in myocytes. These myocytes eventually express mature muscle markers such as MHC, and then start fusing, forming multinucleated myotubes [26].

**Fig. 6:**
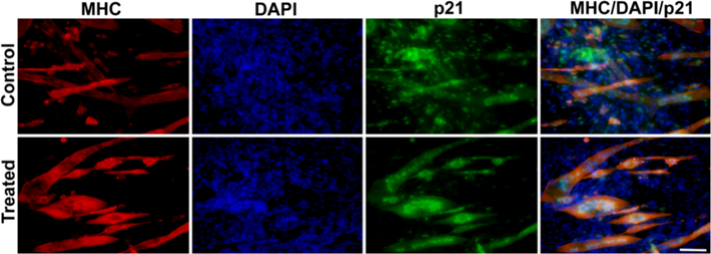
p21 expression is downregulated by treatment with newt extract (see also Table 1). In control cells, p21 expression is seen in myoblasts, as they exit the cell cycle, and in MHC-expressing myocytes. In extract-treated cultures, the extensive p21 expression in mononucleated cells appears to be lost. However, larger myotubes retain p21 expression

C2C12 myoblasts were allowed to differentiate for 5 days. Newt extract in growth medium (GM) was added to the differentiated cells for 3 days followed by immunostaining for p21. A significant decrease in p21 expression was seen in mononucleated cells in treated cultures (Fig. 6 and Table 1, *p* = 0.015). There was also a significant increase (*p* = 0.048) in the p21-/MHC- mononucleated cells (Fig. 6 and Table 1). This suggests that newt extract may block differentiation by blocking p21 expression or may have the ability to downregulate p21 expression in myocytes and thus prevent differentiation and MHC expression.

**Table 1:** A decrease in p21 expression in mononucleated cells following treatment with newt extract

	p21+/MHC+	p21+/MHC−	p21−/MHC−
Control	2.69^a^	11.34	76.4
0.3 mg/ml	2.05	3.42	81.4
*p* value	0.306	0.015	0.048

All nuclei were counted (both in multi-nucleated myotubes and mono-nucleated myocytes and myoblasts), but only mono-nucleated cells are shown here

^a^Percentage of total nuclei

### Cell cycle re-entry is seen in extract-microinjected C2C12 myotubes

Microinjection of extract into multinucleated myotubes (rather than adding extract to the medium) was used as an alternative method of treatment that guaranteed that only differentiated cells were targeted. Myotubes were injected with a combination of extract and GFP plasmid to allow tracking of the injected cells.

**Fig. 7:**
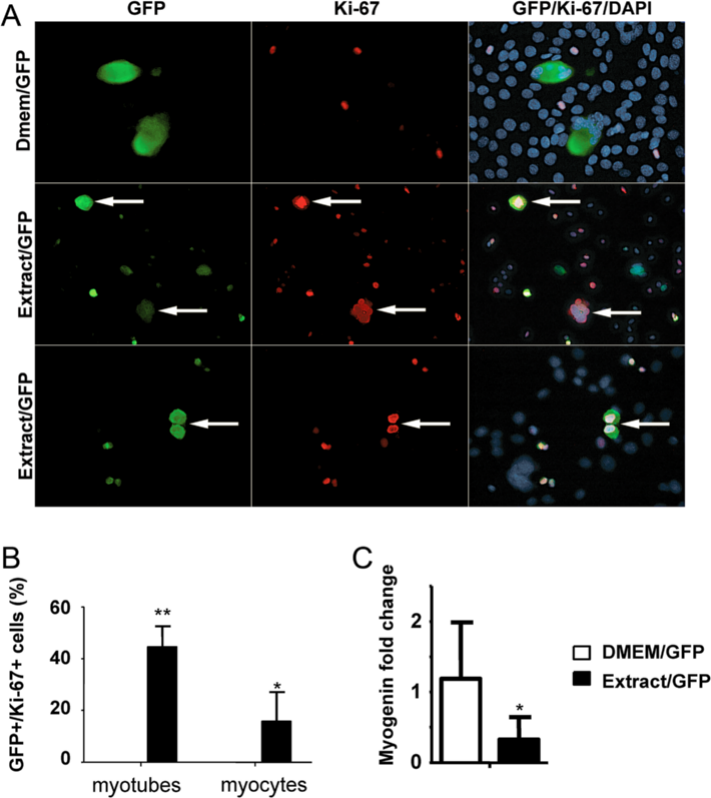
Injection of newt extract directly into C2C12 myotubes. Myotubes were microinjected with either DMEM/GFP (control) or Extract/GFP. **a** *Arrows* identify GFP-positive cells that were also positive for Ki67 (a proliferation marker). **b** Cell counts showed that greater than 40 % of the injected myotubes were positive for Ki67. There were also GFP/Ki67-positive myocytes. Since only multinucleated cells were microinjected, the presence of GFP-positive myocytes suggests that fragmentation may have occurred. **c** RT-PCR of microinjected cultures showed a significant downregulation of myogenin in extract-treated cultures. *p < 0.05, **p < 0.0001

In preparation for microinjection, C2C12 cells were differentiated for 3 days and then filtered. The resulting myotubes were plated on 35-mm plates in DM. Multinucleated cells (of greater than 3 myonuclei) were microinjected with both newt extract and the GFP plasmid (Fig. 7). The control cells were microinjected with the GFP plasmid and Dulbecco’s Modified Eagle Medium (DMEM), which was the vehicle in which the extract was dissolved. As an additional control, we also tested extract isolated from mouse embryonic fibroblasts (MEFs), a rapidly dividing cell line. After microinjection, the cells were transferred to growth medium containing 10 % fetal bovine serum (FBS) for 3 or 4 days. This was followed by staining with Ki-67 (a proliferation marker) and GFP.

In our preliminary studies on microinjection, we compared the results obtained with injection of complete extract with those of fractionated extract (the extract was separated into two fractions: >30 kDa and <30 kDa). These studies showed no cell cycle re-entry in the <30 kDa fraction, and an incomplete response from all nuclei within a myotube for the complete extract. The >30 kDa fraction gave the best results, and so all subsequent microinjection studies were conducted with this fraction.

C2C12 multinucleated myotubes that were injected with both the newt extract and GFP re-entered the cell cycle (Fig. 7). Approximately 45 % of the nuclei in GFP-labeled multinucleated cells exhibited cell cycle re-entry as shown by Ki-67 staining (Fig. 7b). Importantly, this level of cell cycle re-entry was higher than that obtained by incubating the cells with the extract, suggesting that there may be proteins in the extract that impact cell cycle re-entry but are not able to cross the cell membrane. Cell cycle re-entry was not observed in multinucleated cells injected with DMEM and GFP (Fig. 7b) or cells injected with the fractionated MEF extract (data not shown). In addition, mononucleated myocytes which were GFP and Ki-67 positive were only found in cultures injected with the newt extract and GFP (Fig. 7b). Since the microinjection was done in multinucleated cells, the presence of GFP-positive myocytes would suggest that the myotubes were fragmenting. We also examined the levels of expression of myogenin, another differentiation-specific muscle marker. RT-PCR analysis showed a significant downregulation of myogenin in extract-injected cultures (Fig. 7c). Importantly, there was no evidence of apoptosis in any of our cultures, suggesting that the microinjection itself is not toxic to the cells.

### There is no cell cycle re-entry in extract-microinjected primary myotubes

Similar to the C2C12 studies, primary myotubes of three nuclei or more were microinjected with newt extract and GFP. Newt extract did not induce cell cycle re-entry in primary myotubes (data not shown). Cell cycle re-entry was also not observed in the control cells which were injected with DMEM and GFP. Since a number of studies have suggested that the extracellular matrix is critical for dedifferentiation and regeneration in the newt, we assessed whether coating of the plates with collagen or sodium hyaluronate would influence cell cycle re-entry. Neither substrate was able to support cell cycle re-entry in primary myotubes (data not shown).

### Fragmentation of extract-treated C2C12 myotubes

**Fig. 8:**
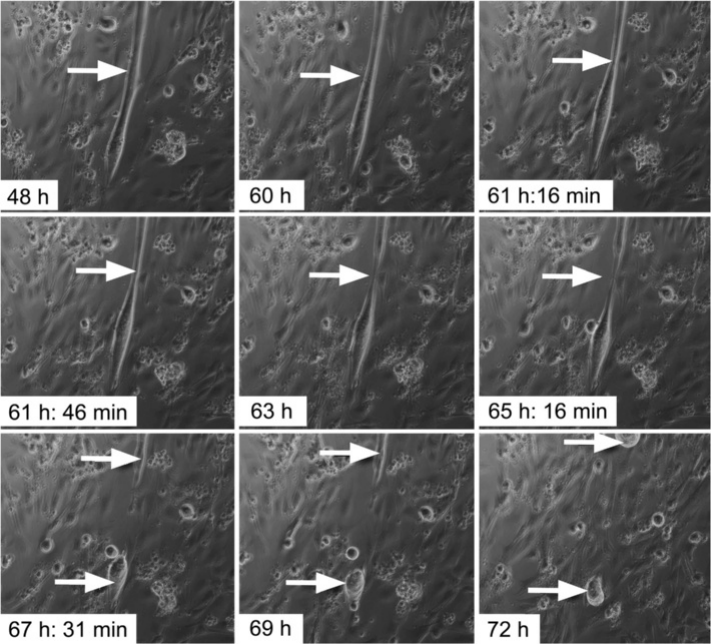
C2C12 myotube (*arrow*) treated with newt extract at a concentration of 0.3 mg/ml (1°) in GM is undergoing fragmentation. The resulting cells remained viable and did not lift off the plate

The above studies suggest that fragmentation of C2C12 multinucleated myotubes is occurring to yield mononucleated myocytes. To confirm this, C2C12 differentiated myotubes were treated with newt extract at 0.3 mg/ml, and followed with Zeiss live imaging microscopy. As shown in Fig. 8, the initial sign of C2C12 myotube fragmentation into two small myotubes was observed after 63 h. After a further 9 h in culture, the fragmented cells were still present in the culture, suggesting that they were still viable.

### Fragmentation of extract-microinjected C2C12 myotubes

To assess whether myotubes microinjected with newt extract can undergo fragmentation and proliferation, C2C12 cells were differentiated for 3 days and then filtered. The filtered cells were re-plated on 35-mm plates coated with 0.4 % collagen. The collagen coating of the plates reduced the degree of cellular migration and allowed better tracking of cells. The following day, the cells were injected with newt extract and GFP plasmid. Control cells were injected with DMEM/GFP. The expression of GFP in myotubes was not detected until at least 24 h after the injection. For that reason, the cells were followed by Zeiss live imaging microscopy starting at 48 h, and images were taken every 15 min. The initial sign of C2C12 myotube fragmentation was observed at approx. 82 h, and the fragmented cells were noticeable as two distinct cells after 90 h (Additional files 6 and 7).

### Fragmentation of extract-microinjected primary myotubes

**Fig. 9:**
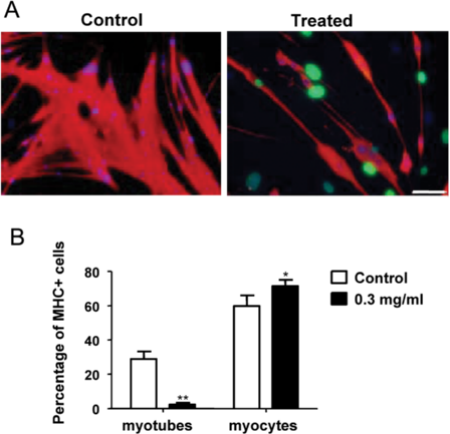
Treatment with newt extract induced fragmentation of primary myotubes. Primary myotubes did not re-enter the cell cycle but were induced to fragment following treatment with 0.3 mg/ml of extract. **a** Visible changes to myotube morphology and structure were seen by treatment with extract. **b** Cell counts showed a significant drop in the number of myotubes and a significant increase in the number of myocytes following treatment with extract. This suggests that fragmentation is occurring

Even though cell cycle re-entry was not seen in primary myotubes treated with the newt extract, we observed that cultures treated with newt extract and GFP had a significant decrease in multinucleated cells and increase in myocytes (one or two nuclei positive for MHC) in comparison to the control (Fig. 9). The decrease in multinucleated cells and the increase in myocytes might indirectly indicate that the newt extract caused fragmentation of multinucleated cells into myocytes. In order to examine this, we tracked injected primary myotubes with the live imaging microscope. In primary cells, the initial sign of fragmentation was seen after 112 h and 35 min (Additional files 8 and 9). By 121 h and 26 min, the cells were fully separated from one another. The cells continued to survive in culture until the experiment was stopped at 263 h and 26 min (data not shown). Myotubes injected with DMEM/GFP did not undergo fragmentation.

## Discussion

Overall, our results show that newt-derived extracts can block the differentiation of both primary and C2C12 myoblasts. Based on our experiments with differentiated C2C12 cultures, we believe that one of the mechanisms by which extract can prevent differentiation of myoblasts may be by preventing the upregulation of p21, which has been shown to cause cell cycle exit during differentiation [26]. A similar inhibition of differentiation was seen by Kim et al. [27] using extract isolated from the blastema of the teleost, *Sternopygus macrurus*. However, the teleost extract was also able to induce proliferation of muscle cells. We did not see a significant proliferation effect by our newt extracts. However, it is noteworthy that Kim et al. treated their cells in differentiation medium (DM), whereas we treated our cells in growth medium (GM). It may be that our cells were already proliferating maximally. We suspect that if we treated our cells in DM, there would probably have been a proliferative effect, similar to the electric fish, since it is inevitable that the newt extracts have growth factors which are required for the proliferation of the dedifferentiated blastema cells. We did not only conduct our proliferation assays in GM but also conducted many of our extract treatments in GM as well. We reasoned that if cells entered the cell cycle, they would need growth factors available to them so that they could proceed through the cell cycle rather than undergoing apoptosis.

Importantly, similar to the results of McGann et al. [18], we found that newt extracts are able to induce cell cycle re-entry and fragmentation of C2C12 myotubes, something which did not occur with the teleost extract. However, the extracts are not able to induce cell cycle re-entry and are only able to induce fragmentation in primary myotubes. In order to ensure that the results we obtained were robust, we used several different methods, including incubation, microinjection, and cell tracking to show that newt extract can induce cell cycle re-entry. All these methods were successful in C12C12 cells but none showed cell cycle re-entry in primary cells. This suggests that C2C12 cells have acquired mutations in culture that may allow them to re-enter the cell cycle. In fact, studies comparing C2C12 cells to primary myoblasts have shown that C2C12 cells have tumorigenic potential [28], have mutations in p19ARF [19], respond differently to p53 [29], and show changes in adhesion protein expression [30]. All of these changes may contribute to their ability to respond to newt extracts. Furthermore, the fact that we only saw cell cycle re-entry in smaller myotubes or myocytes suggests that these cells may be “less differentiated” than the large myotubes and more capable of fragmentation, and this is why they preferentially respond to newt extract. Our studies with p21 would also seem to support this suggestion, as we found no differences in p21 expression in multinucleated myotubes following treatment with newt extract but saw significant downregulation in mononucleated myocytes and myoblasts.

In primary cells, it is possible that we did not identify the ideal conditions to induce cell cycle re-entry. Numerous studies suggest that the extracellular matrix is critical during the dedifferentiation process since it controls tissue stiffness and influences DNA synthesis, cell migration, myotube fragmentation, and myoblast fusion [31, 32]. In our studies, we coated the primary myotube plates with collagen and hyaluronic acid and found no evidence of cell cycle re-entry, although we were able to induce fragmentation. It thus remains a possibility that we simply did not identify the optimal conditions to induce cell cycle re-entry.

Interestingly, we found that not all sources of extract were equally effective. With some sources of extract, the number of cells undergoing cell cycle re-entry were extremely limited (only a few cells in the culture), while other sources of extract showed 25–30 % cell cycle re-entry (and even greater when the extract was microinjected into myotubes rather than added to the culture medium). Coomassie stained Western blots of different batches of extract revealed slight differences in the concentrations (or even presence) of certain proteins. The reasons for this variability are not entirely clear. It may be related to the health of the animals or the variability in the rate of regeneration. In some cases, the animals were clearly smaller and less hearty, and potentially battling other health issues (such as parasitic infections). This was very difficult to control since the newts were wild-caught, and some seasons are better than others in terms of their health and their growth. Overall, only approximately 1/3 of the extracts tested showed cell cycle re-entry. Unfortunately, it was too difficult to conduct all experiments with the same batch of extract since the yield of extract was not large. This disadvantage also served as an advantage because we can conclusively say that the results obtained in this study were not just obtained with one source of extract but with multiple isolates.

In general, we found that secondary extract (i.e., extract obtained from newts undergoing a second round of amputations within 1–3 months of the initial amputations) worked a little better than primary extract. Our best results (highest levels of cell cycle re-entry and lowest levels of toxicity and cell death) were obtained with secondary extract.

One might argue that the reason why newt extracts induced cell cycle re-entry in C2C12 cells was related to the fact that they were isolated from rapidly dividing cells in the early regenerate. This is unlikely since the proliferation of blastema cells is not usually maximal in the early regenerate (within 5 days of amputation). Nevertheless, in order to eliminate the possibility that cell cycle re-entry was induced by proliferation-inducing proteins in the extract, we isolated extract from mouse embryonic fibroblasts during their exponential growth. These extracts had no effects on cell cycle re-entry, suggesting that the cell cycle-inducing property is newt cell-specific.

It is interesting to note that treatment with the cytosine analog AraC induced death in myotubes (especially larger myotubes with greater numbers of nuclei). While we saw some evidence of this even in our control cultures, it was much more pronounced in the cultures treated with a combination of AraC and newt extracts. We believe that the explanation for this may be the following: Cultures were treated with AraC to allow incorporation of the nucleotide analog into the DNA of cycling myoblasts. This would normally lead to impaired DNA replication and death of the myoblasts, yielding a purer myotube population. We believe the AraC was also taken up by myotubes. Even if the cultures were then washed to remove excess AraC prior to extract treatment, any AraC that was retained in the cells could become incorporated into DNA if the cells were subsequently induced to undergo cell cycle re-entry by the extract treatment. Once this occurred, this would lead to the death of the myotubes. For this reason, we switched from AraC treatment to filtration in later experiments to eliminate contaminating myoblasts from the myotube cultures.

## Conclusions

Overall, we have shown that newt extracts can induce cell cycle re-entry of C2C12 myotubes, but they only induced fragmentation of primary myotubes. It is interesting to note that C2C12 cells have been previously shown to re-enter the cell cycle following knockdown of Rb, but that in primary cells, both Rb and p19ARF knockdown are required to induce cell cycle re-entry [19]. This suggests that C2C12 cells are in an intermediate state between newt A1 muscle cells (which can re-enter the cell cycle following serum stimulation) and primary cells, which require two mutation events for cell cycle re-entry to occur.

## Additional files

Additional file 1:
**Procedure for isolation of newt extract.** The primary (1 °) newt extract was collected by first amputating above the wrist of the newt forelimb to remove the intact tissue. After 5 days, the growing regenerating pre-blastema was removed and frozen in liquid nitrogen. After 3 days the limb was re-amputated, and then re-amputated again the following day. The 1, 3 and 5-day regenerates were pooled. The secondary (2°) extract was prepared from animals that had been previously amputated, and allowed to regenerate for 1–3 months. Pooled tissues were subjected to homogenization and centrifugation as described in the “Methods” section. Additional file 2:
**Extract treatment of subconfluent myoblasts did not affect proliferation.** A) Flow cytometry profiles for control C2C12 cells, or C2C12 cells treated with 0.3 mg/ml of newt extract showed no visible differences between the two groups. B) Values obtained from flow analysis showing the percentage of cells in each of the cell cycle compartments appeared similar between control and extract-treated cells. C) Cell counts analyzed with a hemocytometer agreed with flow cytometry results. Values are shown +/− Standard Deviation. Additional file 3:
**Coomassie blue-stained western blot of four different extract isolates.** Coomassie blue-stained western blot of four different extract isolates (2 primary and 2 secondary extracts) shows that the protein pattern is not identical between different batches of extract. This may be the reason why some batches were more effective than others in inducing cell cycle re-entry. Additional file 4:
**Cell cycle re-entry was not seen in primary myotube cultures.** Myotube cultures were treated with AraC to eliminate cycling myoblasts, and then treated with various concentrations of 1 ° or 2 ° newt extract in the culture medium. Cultures were stained with MHC (green), BrdU (red) and DAPI (nuclear stain). A) Control cultures showed no BrdU incorporation in MHC-expressing cells. B) Extract-treated myotubes also typically showed no BrdU incorporation. The BrdU positive nucleus (arrow) in the myotube was the only one seen in all cultures treated, and probably resulted from a cycling myoblast fusing with a pre-existing myotube. Additional file 5:
**The effects of AraC treatment on myotubes.** A) TUNEL assay identified apoptotic cells in AraC treated C2C12 differentiated cells. The higher the concentration of extract, the more toxicity was seen in myotubes. Cell death was only seen in extract treatment combined with AraC. B) Comparison of AraC treated and non-AraC-treated myotube cultures showed fewer and smaller myotubes in the AraC and extract-treated cultures, suggesting that the combination of AraC and extract caused death of larger myotubes. Each experiment was performed in triplicate. *p < 0.0001 between the control and cultures treated with 0.3 mg/ml. Additional file 6:
**Fragmentation in C2C12 myotubes treated with newt extract.** C2C12 myotube (with what appeared like 3–4 nuclei), injected with newt extract can undergo fragmentation. The arrows are pointing to the same myotube as it is breaking down into two cells. The images were taken every 20 mins using a Zeiss live imaging microscope. The time shown is when the image was taken. D) inset, shows an enhanced image of the cell (with 3–4 nuclei) prior to its fragmentation (see accompanying time-lapse video (Additional file 7)). Additional file 7:
**Time lapse video of fragmenting C2C12 myotube. **Time lapse video of the images portrayed in Additional file 6 showing fragmentation of a multinucleated C2C12 myotube. Additional file 8:
**Treatment with newt extract induced fragmentation of a primary myotube.** Myotube cultures were tracked with live imaging (see also time-lapse video (Additional file 9). The arrowheads point to a GFP- and extract-injected myotube as it undergoes fragmentation. The black arrowheads point to two cells originating from a single myotube as they pull apart. The white arrowhead identifies a third cell which also appears to originate from the myotube, but may potentially be a cell which was above the plane of the myotube in (B). Additional file 9:
**Time-lapse video of fragmenting primary myotube.** Time-lapse video of the images portrayed in Additional file 8 showing fragmentation of a multinucleated primary myotubes.

## Abbreviations

AraC: arabinosylcytosine
BrdU: bromodeoxyuridine
DM: differentiation medium
DMEM: Dulbecco’s Modified Eagle Medium
GFP: green fluorescent protein
GM: growth medium
MCK: muscle creatine kinase
MEF: mouse embryonic fibroblasts
MHC: myosin heavy chain
RFP: red fluorescent protein
TUNEL: terminal deoxynucleotidyl transferase (TdT)-mediated dUTP nick end labeling
