# Supplementary figures and images for: A differential response to newt regeneration extract by C2C12 and primary mammalian muscle cells
Source: Skelet Muscle. 2015 Jun 11;5:19. doi: 10.1186/s13395-015-0044-8 (PMC4471912; doi:10.1186/s13395-015-0044-8)

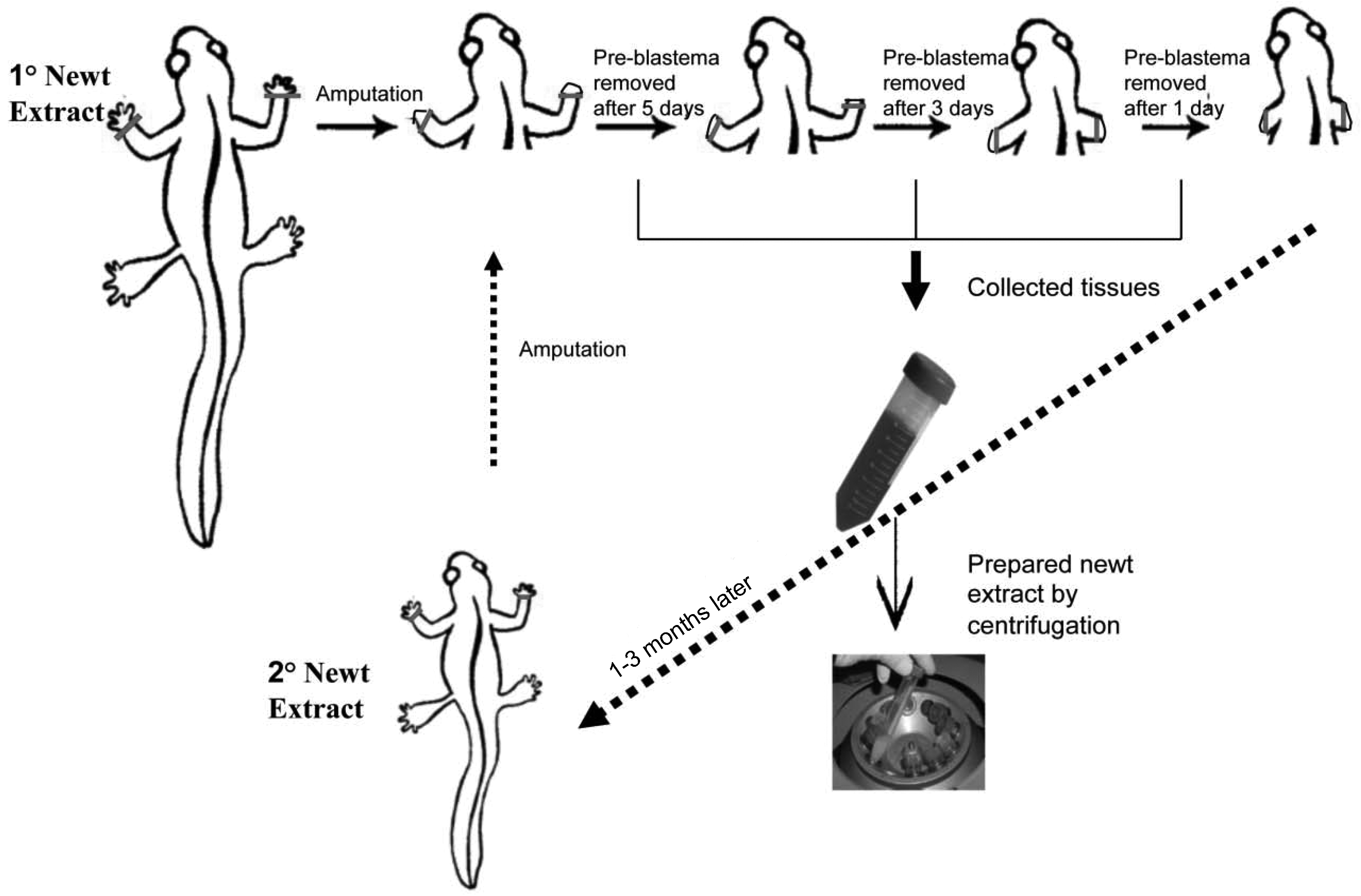

Supplement: Additional file 1: — Procedure for isolation of newt extract. The primary (1 °) newt extract was collected by first amputating above the wrist of the newt forelimb to remove the intact tissue. After 5 days, the growing regenerating pre-blastema was removed and frozen in liquid nitrogen. After 3 days the limb was re-amputated, and then re-amputated again the following day. The 1, 3 and 5-day regenerates were pooled. The secondary (2°) extract was prepared from animals that had been previously amputated, and allowed to regenerate for 1–3 months. Pooled tissues were subjected to homogenization and centrifugation as described in the “Methods” section. [file 13395_2015_44_MOESM1_ESM.tiff]

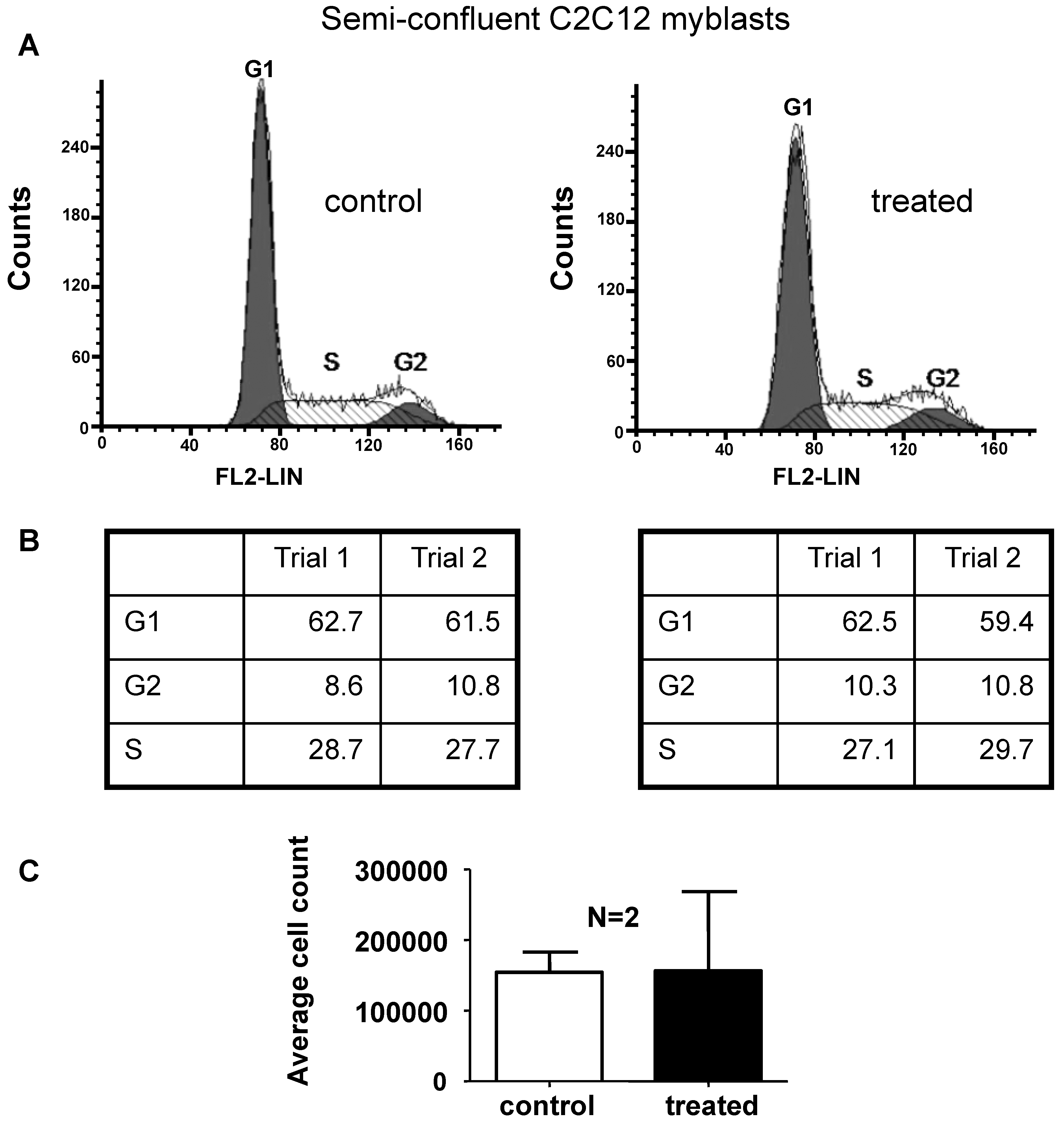

Supplement: Additional file 2: — Extract treatment of subconfluent myoblasts did not affect proliferation. A) Flow cytometry profiles for control C2C12 cells, or C2C12 cells treated with 0.3 mg/ml of newt extract showed no visible differences between the two groups. B) Values obtained from flow analysis showing the percentage of cells in each of the cell cycle compartments appeared similar between control and extract-treated cells. C) Cell counts analyzed with a hemocytometer agreed with flow cytometry results. Values are shown +/− Standard Deviation. [file 13395_2015_44_MOESM2_ESM.tiff]

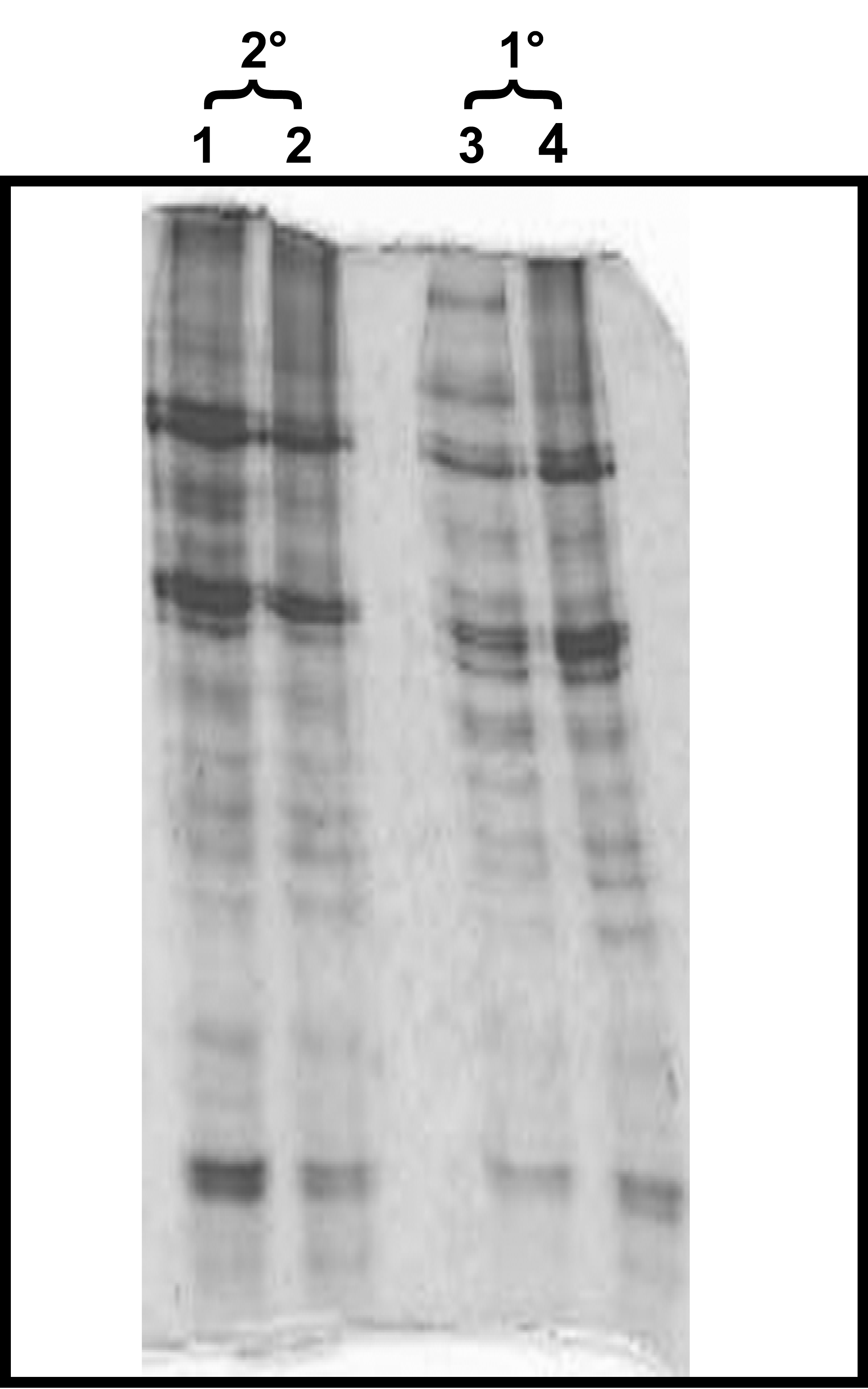

Supplement: Additional file 3: — Coomassie blue-stained western blot of four different extract isolates. Coomassie blue-stained western blot of four different extract isolates (2 primary and 2 secondary extracts) shows that the protein pattern is not identical between different batches of extract. This may be the reason why some batches were more effective than others in inducing cell cycle re-entry. [file 13395_2015_44_MOESM3_ESM.tiff]

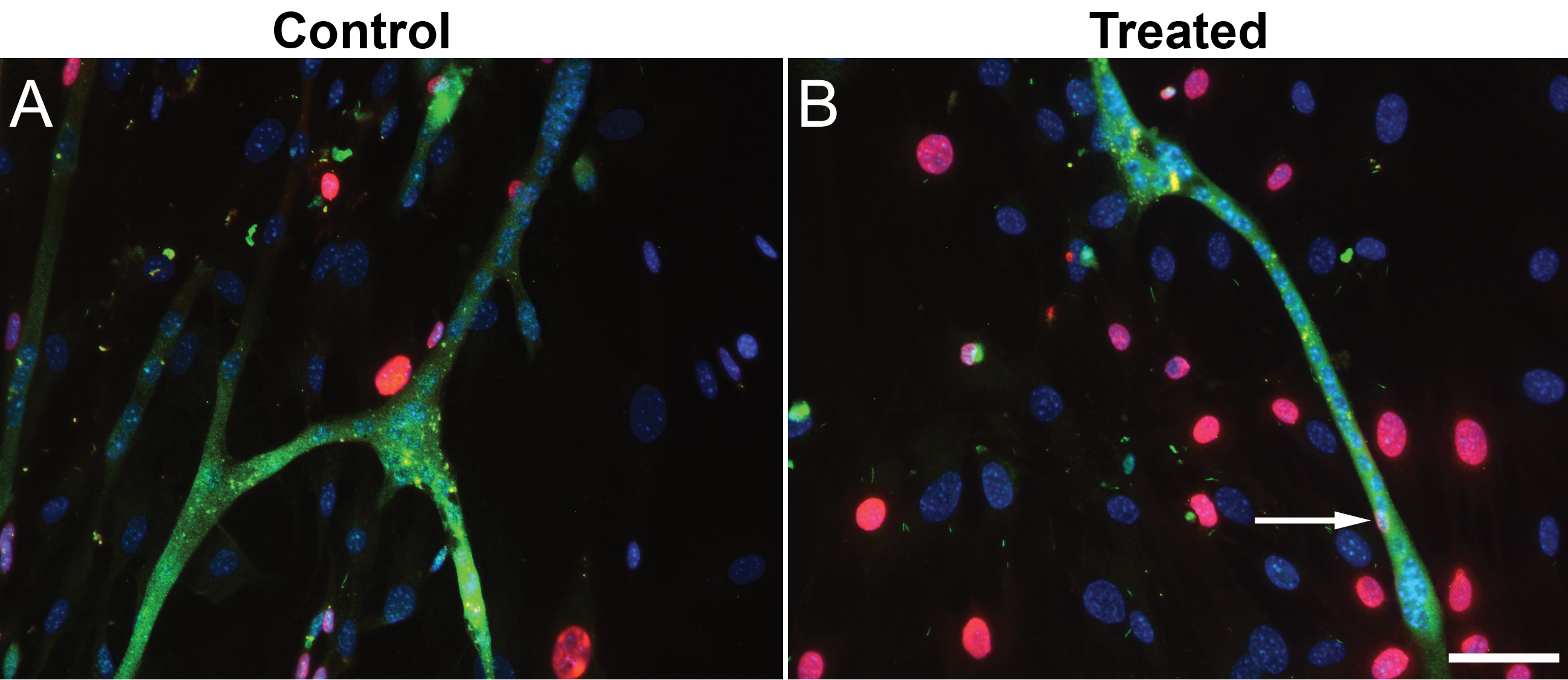

Supplement: Additional file 4: — Cell cycle re-entry was not seen in primary myotube cultures. Myotube cultures were treated with AraC to eliminate cycling myoblasts, and then treated with various concentrations of 1 ° or 2 ° newt extract in the culture medium. Cultures were stained with MHC (green), BrdU (red) and DAPI (nuclear stain). A) Control cultures showed no BrdU incorporation in MHC-expressing cells. B) Extract-treated myotubes also typically showed no BrdU incorporation. The BrdU positive nucleus (arrow) in the myotube was the only one seen in all cultures treated, and probably resulted from a cycling myoblast fusing with a pre-existing myotube. [file 13395_2015_44_MOESM4_ESM.tiff]

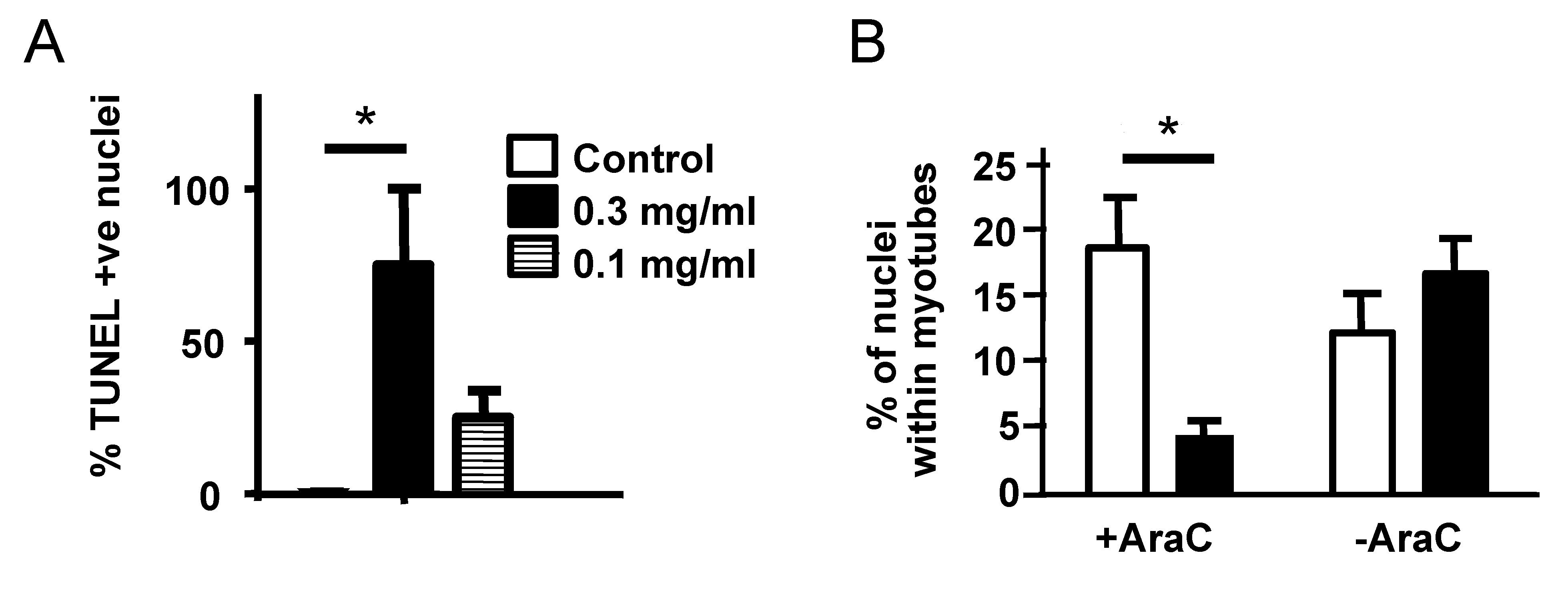

Supplement: Additional file 5: — The effects of AraC treatment on myotubes. A) TUNEL assay identified apoptotic cells in AraC treated C2C12 differentiated cells. The higher the concentration of extract, the more toxicity was seen in myotubes. Cell death was only seen in extract treatment combined with AraC. B) Comparison of AraC treated and non-AraC-treated myotube cultures showed fewer and smaller myotubes in the AraC and extract-treated cultures, suggesting that the combination of AraC and extract caused death of larger myotubes. Each experiment was performed in triplicate. *p < 0.0001 between the control and cultures treated with 0.3 mg/ml. [file 13395_2015_44_MOESM5_ESM.tiff]

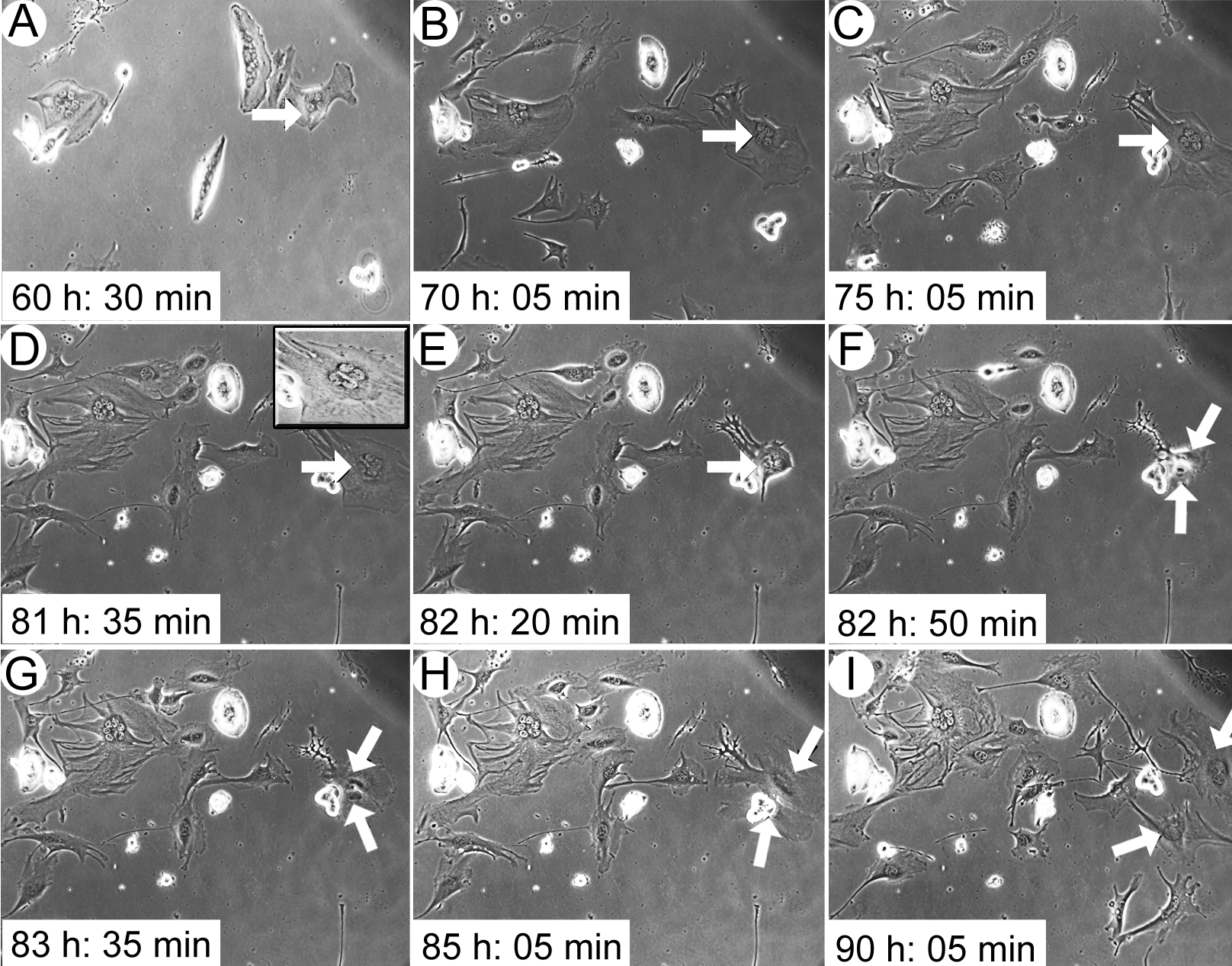

Supplement: Additional file 6: — Fragmentation in C2C12 myotubes treated with newt extract. C2C12 myotube (with what appeared like 3–4 nuclei), injected with newt extract can undergo fragmentation. The arrows are pointing to the same myotube as it is breaking down into two cells. The images were taken every 20 mins using a Zeiss live imaging microscope. The time shown is when the image was taken. D) inset, shows an enhanced image of the cell (with 3–4 nuclei) prior to its fragmentation (see accompanying time-lapse video (Additional file 7)). [file 13395_2015_44_MOESM6_ESM.tiff]

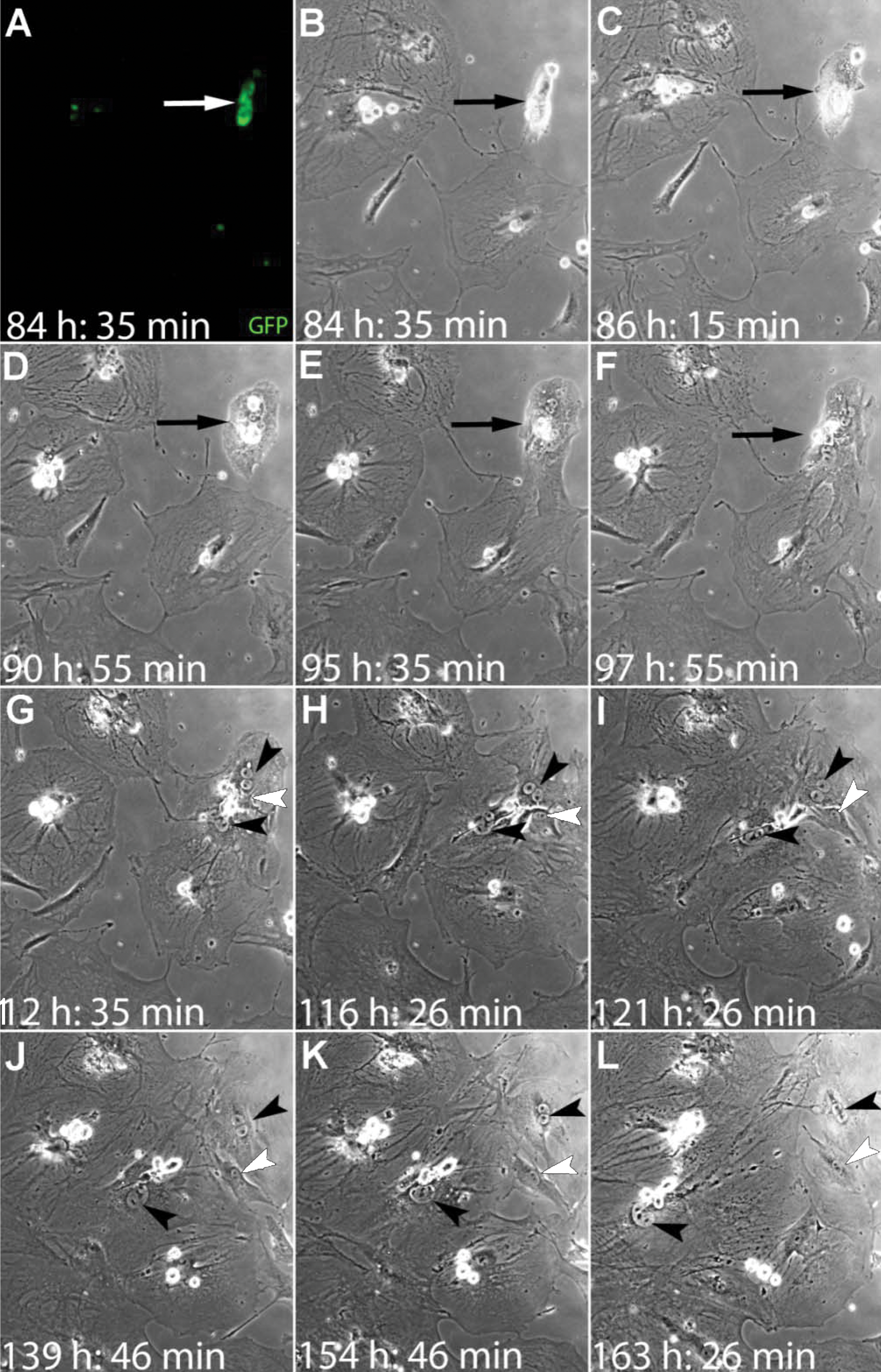

Supplement: Additional file 8: — Treatment with newt extract induced fragmentation of a primary myotube. Myotube cultures were tracked with live imaging (see also time-lapse video (Additional file 9). The arrowheads point to a GFP- and extract-injected myotube as it undergoes fragmentation. The black arrowheads point to two cells originating from a single myotube as they pull apart. The white arrowhead identifies a third cell which also appears to originate from the myotube, but may potentially be a cell which was above the plane of the myotube in (B). [file 13395_2015_44_MOESM8_ESM.tiff]
